# Analysis of the receptor BCMA as a biomarker in systemic lupus erythematosus patients

**DOI:** 10.1038/s41598-020-63390-0

**Published:** 2020-04-10

**Authors:** Diana Celeste Salazar-Camarena, Claudia Azucena Palafox-Sánchez, Alvaro Cruz, Miguel Marín-Rosales, José Francisco Muñoz-Valle

**Affiliations:** 1 Research Institute in Biomedical Sciences, University Center of Health Sciences, University of Guadalajara, Guadalajara, Mexico; 2 Department of Rheumatology, West Medical Hospital, Ministry of Health, Zapopan, Mexico

**Keywords:** Immunology, Prognostic markers, Rheumatology

## Abstract

B cell activating factor (BAFF) and a proliferation-inducing ligand (APRIL) play central roles in B cell development and maturation. Soluble forms of their receptors can be generated by proteolytic cleavage; however, their physiological and clinical roles are unknown. This study aimed to assess the relationships between the receptor soluble B cell maturation antigen (sBCMA) and clinical variables in systemic lupus erythematosus (SLE) patients. Serum cytokine concentrations were measured by ELISA for 129 SLE patients and 34 healthy controls (HCs), and the expression of the receptor BCMA was evaluated on B and plasma cells from 40 subjects. SLE patients showed aberrant expression of the receptor BCMA on B and plasma cells. Soluble levels of the receptor sBCMA and its ligands sAPRIL and sBAFF were increased in SLE patients compared with HCs. Additionally, sBCMA (r_s_ = 0.6177) and sAPRIL (r_s_ = 0.4952) correlated strongly with disease activity. Active SLE patients who achieved low disease activity showed decreased sBCMA (53.30 vs 35.30 ng/mL; *p* < 0.05) and sBAFF (4.48 vs 2.27 ng/mL; *p* < 0.05) serum levels after treatment, while sAPRIL expression remained unchanged. At a cutoff value of 22.40 ng/mL, sAPRIL showed high sensitivity (96.12%) and specificity (94.12%) for discrimination between HCs and SLE patients, while sBAFF showed lower sensitivity (82.2%) but higher specificity (94.1%) at a cutoff of 1.195 ng/mL. Relatively high levels of sAPRIL and sBCMA clustered active SLE patients. The receptor sBCMA could be a potential biomarker of disease activity in SLE.

## Introduction

Systemic lupus erythematosus (SLE) is a chronic autoimmune disease caused by perturbations in the immune system. The extreme heterogeneity of this disease is primarily explained because genetic background confers susceptibility and environmental factors act as triggers that contribute to disease initiation and progression^1^. A key point in SLE pathogenesis is an imbalance between apoptotic cell numbers and apoptotic material disposal that leads to activation of the humoural response. The serological hallmark of SLE is the production of autoantibodies, which target antigens located in the nucleus or destined for the cell surface in the cytoplasm and are secreted by cells^2^. As T and B cell abnormalities are thought to be central to the disease process^3^, the cytokines that promote B cell differentiation and loss of tolerance have emerged as essential players in the pathophysiology of SLE^1,4^. In the regulation of B cell activation, several members of the tumour necrosis factor (TNF) superfamily participate, including B cell activating factor (BAFF) and A Proliferation-Inducing Ligand (APRIL). These stimulating factors play central roles in B cell development and maturation^5^. Altered serum levels of these cytokines have been found in autoimmune diseases such as SLE^6–11^, rheumatoid arthritis^12,13^ and Sjögren’s syndrome^14,15^. Three BAFF and APRIL receptors have been described: BCMA (B-cell Maturation Antigen), TACI (Transmembrane Activator and CALM Interactor) and BAFF-R (also known as BAFF receptor or BR3), which constitute the BAFF/APRIL system^16^. Recent studies have found these receptors to be soluble isoforms that act as decoy receptors^17^, controlling B cell survival and differentiation. However, their roles in SLE pathogenesis are far from clear. BCMA, also known as TNF receptor superfamily member 17 (TNFRSF17) or CD269, is a receptor that was first identified in a T cell tumour line and later reported to be expressed in B cell lines and immune organs^18^. In the periphery, BCMA is expressed mainly in terminally differentiated B cells^19^, but its expression is not restricted to normal tissues. Furthermore, multiple myeloma (MM) cells^20^, chronic lymphocytic leukaemia cells^21^ and the fibroblast-like synoviocytes of RA patients^22^ express BCMA. Upon stimulation by its ligands, BCMA activates MAP kinases and induces anti-apoptotic proteins, such as Bcl-2 and Bcl-XL^23^, leading to signals promoting cell survival and proliferation. The participation of BCMA in the BAFF/APRIL system has remained elusive. The activation of B cells induces the expression of BCMA, which is accompanied by a reduction in BAFF-R expression^24^. Regarding expression at the peripheral level, the expression of BCMA is increased on the cell surface of late-stage cells, such as memory B cells and plasma cells^25^. It seems that under physiological conditions, BCMA is not required for B cell maturation; however, it is an essential receptor for sustaining enduring antibody protection by mediating the survival of long-lived plasma cells^26^. Kim *et al*. demonstrated that B cells isolated from SLE patients upregulated BCMA expression after TLR9 stimulation, which led to antinuclear antibody (ANA) secretion^25^. These observations suggest that autoantigens derived from nuclear material can contribute to enhanced autoantibody production through BCMA. Recently, a research group demonstrated that BCMA could be recognized through the cysteine-rich domain (CRD) and underwent direct shedding mediated by the γ-secretase enzyme, releasing the soluble receptor form^27^. Elevated sBCMA expression is found in multiple myeloma^28^ and primary central nervous system lymphoma patients^21^. In the context of autoimmune diseases, sBCMA expression is significantly increased in RA^12^ and SLE patients^29^ and correlates with disease activity^27^. As the participation of sBCMA in SLE has been poorly explored despite the relevant role of the BAFF/APRIL system in this disease, the main aim of this study was to analyse the profiles of the B cell factor sBCMA and its ligands sBAFF and sAPRIL in Mexican SLE patients to evaluate the clinical relevance of these molecules.

## Results

### Patient characteristics

The general characteristics and primary demographics of all enrolled participants including 129 SLE patients (123 females and six males) and 34 healthy controls are summarized in Table 1. The clinical variables of the SLE patients were as follows: median activity index of 6 (range =  0–32) and median chronicity index of 0 (range =  0–7). The primary clinical manifestations were haematological (72%), mucocutaneous (50%), and musculoskeletal (30%).

**Table 1:** Demographic and clinical parameters of HCs and SLE patients.

	SLE (n = 129)	HC (n = 34)	*p*
*Demographical features*			
Age, years^a^; median (range)	33 (18–74)	31 (21–59)	0.069
Gender, (F/M)^b^	123/6	33/1	1.000
*Disease features*			
Disease duration, years; median (range)	5 (0–27)		
Mex-SLEDAI score; median (range)	3 (0–20)		
SLEDAI-2K score; median (range)	6.0 (2.0–11.75)		
SLICC score; median (range)	0 (0–7)		
*Clinical manifestations*			
Haematological, *n (%)*	72 (55.8%)		
Mucocutaneous, *n (%)*	50 (39.1%)		
Musculoskeletal, *n (%)*	30 (23.3%)		
Renal disorder, *n (%)*	29 (22.5%)		
Serous, *n (%)*	10 (7.8%)		
Neuropsychiatric, *n (%)*	2 (1.6%)		
*Treatment*			
Prednisone, *n* (%)	97 (75.8%)		
Azathioprine, *n* (%)	72 (56.3%)		
Antimalarial, *n (%)*	64 (50.0%)		
Cyclophosphamide, *n* (%)	21 (16.4%)		
Methotrexate, *n* (%)	25 (19.5%)		
*Laboratory data*			
ESR (mm h^−1^); median (p25- p75)	32 (16.0–49.0)	12 (6.75–16.0)	**<0.001**
ANA + ve (>1280), *n (%)*	49 (40%)		
Anti-dsDNA + ve, *n (%)*	62 (48.1%)		
Anti-dsDNA (UI/mL); median (p25- p75)	20.89 (9.27–63.21)		
C3 (mg/dL); median (p25- p75)	94.95 (75.37–136.8)		
C4 (mg/dL); median (p25- p75)	24.05 (18.6–36.82)		
Proteinuria (≥500 mg/day), *n (%)*	30 (23.26%)		
*Soluble B-cell factors*			
sBCMA^a^, ng/mL; media±SD median (p25- p75)	51.89 ± 23.69 49.03 (33.08–63.8)	25.44 ± 6.028 25.60 (21.63–29.19)	**<0.001**
sBAFF^a^,ng/mL; media±SD median (p25- p75)	3.14 ± 3.07 2.155 (1.47–3.82)	0.84 ± 0.19 0.79 (0.69–0.96)	**<0.001**
sAPRIL^a^, ng/mL; media±SD median (p25- p75)	33.66 ± 21.41 28.02 (17.50–49.02)	10.47 ± 13.15 8.23 (3.51–12.68)	**<0.001**

Data are shown as the median and p25 – p75. Mex-SLEDAI: Mexican version of the Systemic Lupus Erythematosus Disease Activity Index; SLEDAI-2K: Systemic Lupus Erythematosus Disease Activity 2000 Index; SLICC: Systemic Lupus International Collaborating Clinics. ^a^ Mann-Whitney U test. ^b^ Exact Fisher test.

### mBCMA expression is decreased in the B cells of SLE patients

**Figure 1:**
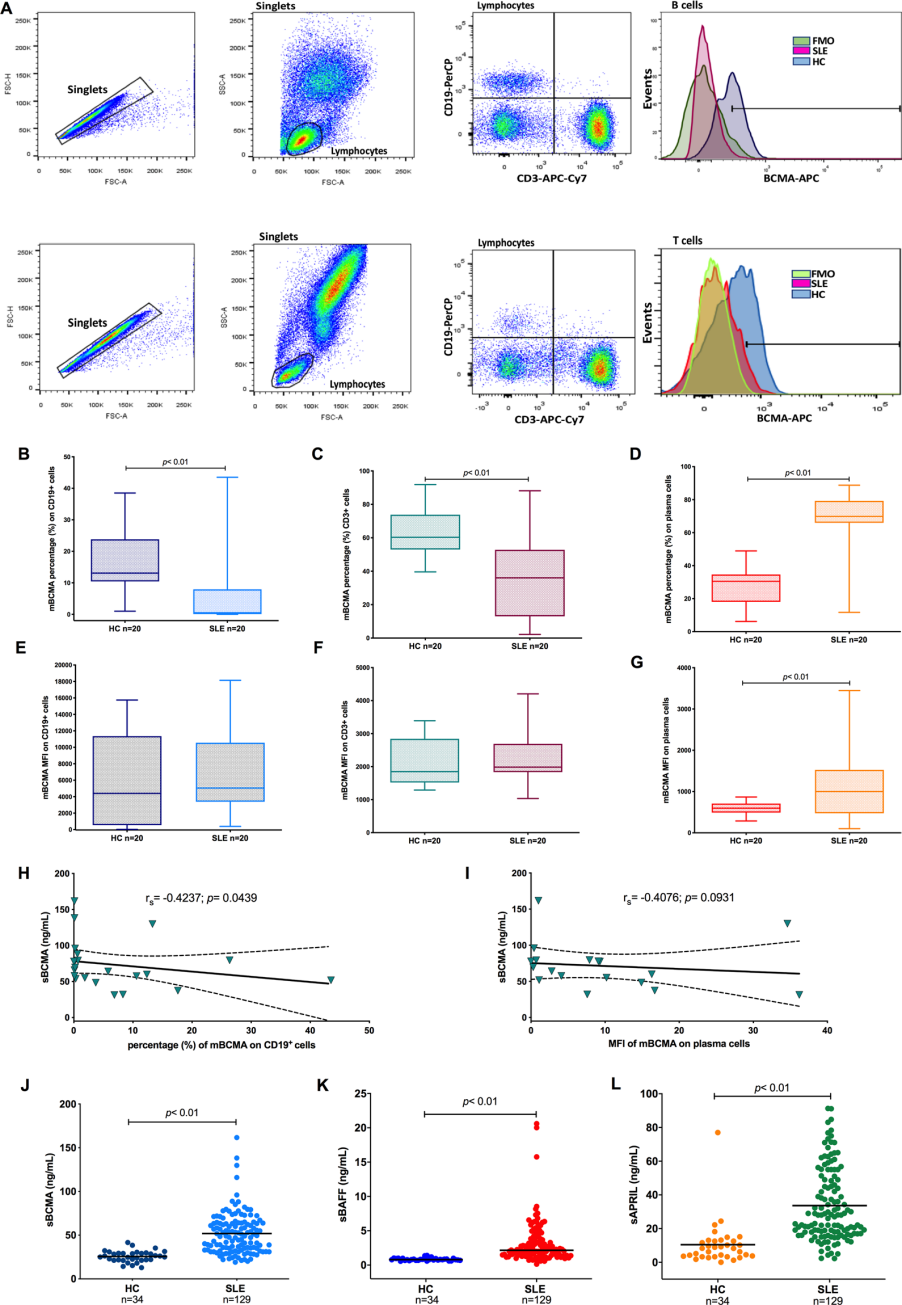
Distribution of BCMA in SLE patients and HCs. (**A**) Gating strategy example for BCMA on CD3+ and CD19+ cells. The percentages of CD19+ cells (**B**), CD3+ cells (**C**) and plasma cells (**D**) from SLE patients or HCs with membrane BCMA (mBCMA) expression. mBCMA MFIs of CD19+ cells (**E**), CD3+ cells (**F**) and plasma cells (**G**) from SLE patients or HCs. Correlations between the level of sBCMA and mBCMA+ percentage of CD19+ cells (**H**) or mBCMA MFI of plasma cells (**I**). Serum concentrations (ng/mL) of the cytokines (**J**) B-cell maturation antigen (BCMA), (**K**) B cell activating factor (BAFF), and (**L**) a proliferation-inducing ligand (APRIL). The bars show the median level, and the error bars represent the min-max. Statistical analysis was performed using the Mann-Whitney *U* test.

BCMA receptor expression was decreased in the CD19^+^ B cells of SLE patients compared with those of HCs (median: 0.5% vs 13.10%, respectively; *p* < 0.01) (Fig. 1B) and in CD3+ cells (median: 36.00% vs 60.10%, respectively; p < 0.01) (Fig. 1C), while in plasma cells, the percentage of positive cells was increased in the SLE group (median of 69.85) compared with the HC group (median of 30.50; *p* < 0.01) (Fig. 1D). Additionally, the levels of soluble BCMA were inversely correlated with the percentage of mBCMA^+^ CD19^+^ B cells in SLE patients (r_s_ = −0.4237, *p* < 0.05) (Fig. 1H).

### sBCMA expression is elevated in SLE patients

**Figure 2:**
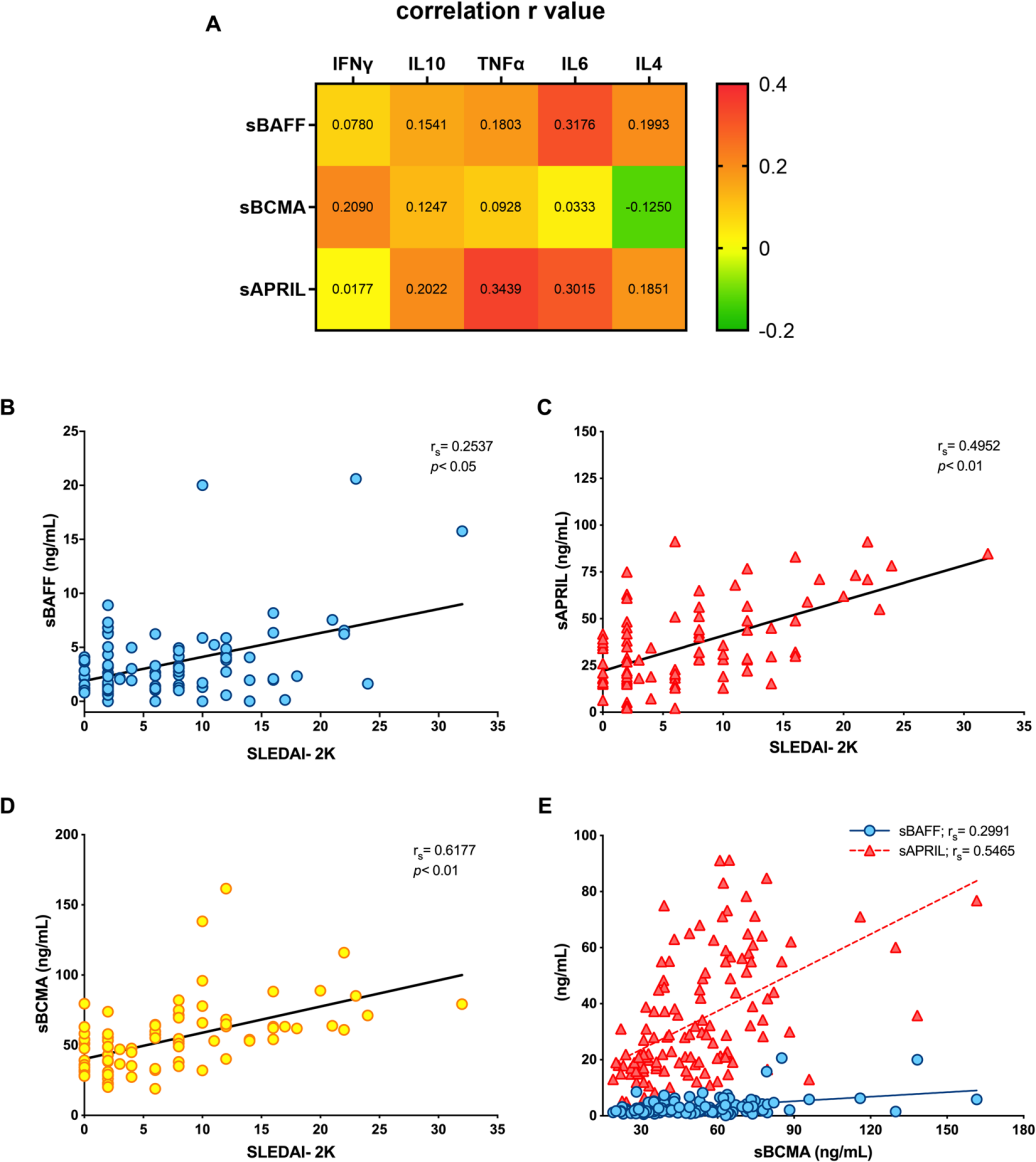
Correlation matrix heatmap (**A**) of soluble B cell proliferation factors and the cytokines IFNγ, IL10, TNFα, IL6 and IL4. (**B–D**) Associations of soluble B cell factor levels and disease activity in SLE patients. r_s_: Spearman´s correlation coefficient, SLEDAI-2K: Systemic Lupus Erythematosus Disease Activity 2000 Index.

The level of the decoy receptor sBCMA was elevated in SLE patients (49.03 ng/mL) compared with HCs (25.60 ng/mL; *p* < 0.05) (Fig. 1J). The levels of the B cell factors sBAFF (median: 2.15 vs 0.79 ng/mL, respectively; *p* < 0.05) and sAPRIL (median: 28.02 vs 8.23 ng/mL, respectively; *p* < 0.05) were elevated in SLE patients compared with HCs (Fig. 1K,L). sBCMA moderately correlated with IFNγ (r = 0.2090, *p* = 0.034), while sBAFF correlated with the serum level of IL6 (r_s_ = 0.3176, *p* = 0.001). Last, sAPRIL correlated with the serum levels of IL10 (r_s_ = 0.2022, *p* = 0.041), TNFα (r_s_ = 0.3439, *p* = 0.004), and IL6 (r_s_ = 0.3015, *p* = 0.002) (Fig. 2A).

### Correlations with a disease activity index

As shown in Fig. 2B, sBAFF had a modest correlation (r_s_ = 0.2537) with disease activity, while both sAPRIL (r_s_ = 0.4952) and sBCMA (r_s_ = 0.6177) showed a strong correlation with disease activity (Fig. 2C,D). The serum levels of sBCMA correlated with those of both ligands. However, the correlation was stronger with sAPRIL (r_s_ = 0.5465) than with sBAFF (r_s_ = 0.2991), probably indicating preferential binding in the periphery (Fig. 2E). The serum levels of APRIL (r_s_ = 0.3998) and BCMA (r_s_ = 0.2789) correlated with the SLICC damage index. Increased sBCMA expression was found in anti-dsDNA antibody-positive patients (58.00 vs 39.17 ng/mL; *p* < 0.01) and active LN patients (64.82 vs 41.25 ng/mL; *p* < 0.01) (Supplementary Fig. S1).

### sBCMA and sBAFF levels in an SLE cohort

**Figure 3:**
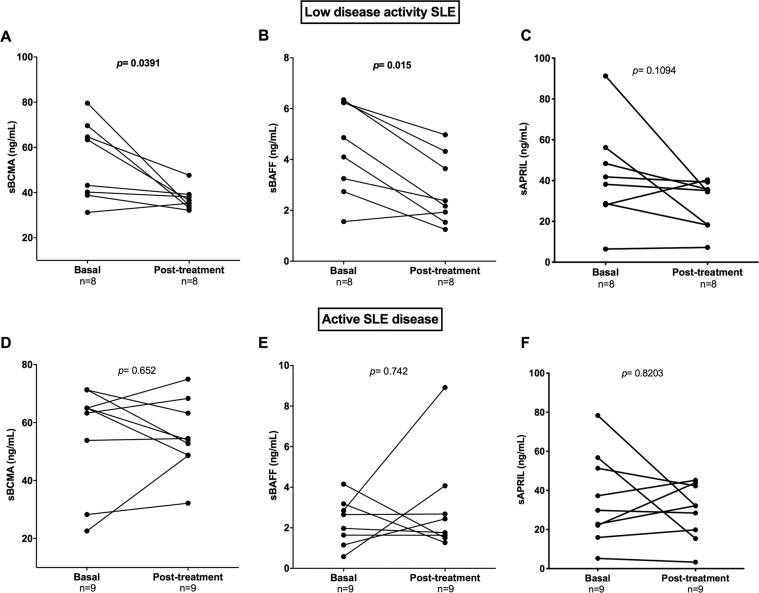
Trends in serum B cell proliferation factor levels in SLE patients after 6 months of conventional treatment. SLE patients were classified into the low disease activity (LDA) and active disease (AD) groups according to their SLEDAI-2K score after conventional treatment. Statistical analysis was performed using Wilcoxon’s matched‐pairs signed‐rank test.

**Figure 4:**
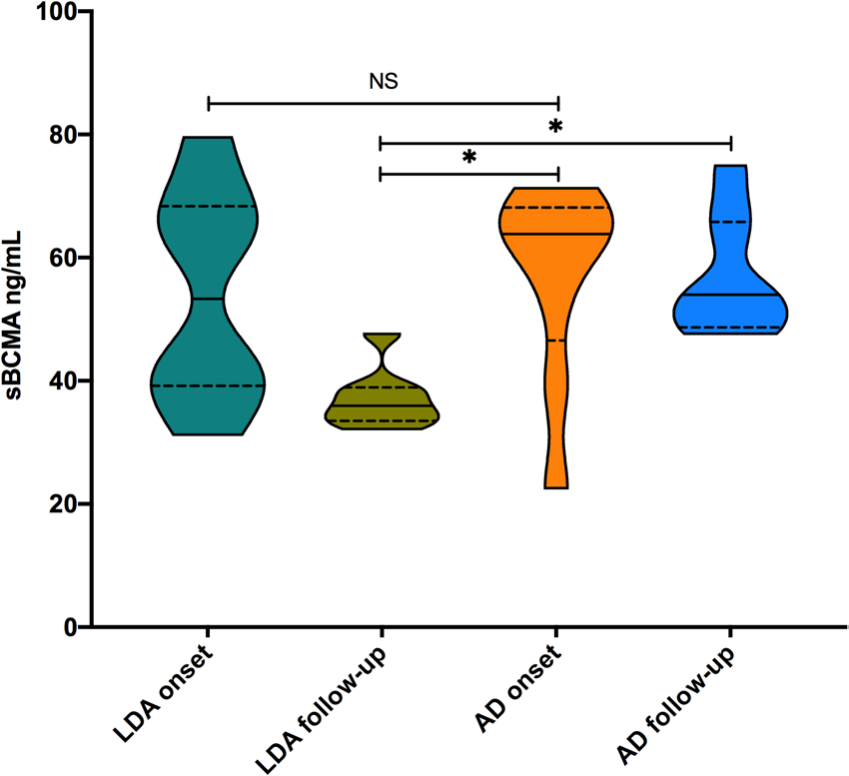
sBCMA levels among HCs and SLE patients with low disease activity (LDA) or active disease (AD) after 6 months of conventional treatment. Kruskal-Wallis test with Dunn’s multiple comparisons test. *p < 0.05. The black line depicts the median, and the dotted line shows p25-p75.

Seventeen SLE patients were included in a prospective study to evaluate changes in soluble BAFF, APRIL and BCMA levels and associate these changes with clinical manifestations (Supplementary Table). Patients received standard-of-care therapy according to disease manifestations evaluated during clinic visits as scheduled by the treating physician. In the prospective group, eight out of the 17 SLE patients (47.1%) achieved an LDA state (defined as the Lupus Low Disease Activity State^30^) at the end of 6 months of therapy. On the other hand, SLE patients who showed increased scores or remained active were classified as the active disease (AD) group. Figure 3A,B shows that the patients in the LDA group achieved decreased sBCMA levels between visits (median: 53.30 vs 35.94 ng/mL; *p* = 0.039) as well as reduced sBAFF levels (4.48 vs 2.27 ng/mL; *p* = 0.015).The AD SLE group exhibited maintenance of similar serum levels of sBCMA and their ligands between visits and showed no significant differences (Fig. 3D–F). According to the BILAG index, the major affected domains were haematological (94%), renal (76%), musculoskeletal (76%) and mucocutaneous (59%). As observed, patients with the haematological or renal domain affected maintained clinical activity or relapsed during follow-up (56% and 61%), respectively, while the majority of clinical improvements were observed in patients with the musculoskeletal (76.9%) or mucocutaneous domain affected. With the available data, we analysed whether sBCMA identifies patients that achieve LDA during follow-up using logistic regression analysis, and the overall percentage was 82.4%, with an OR of 0.93 (95% CI: 0.873–1.004) that was nonsignificant. The basal levels of sBCMA in the LDA and AD SLE patients were not different (median: 53.30 vs 63.85 ng/mL, respectively) (Fig. 4).

### Predictive usefulness of soluble B cell proliferation factors as biomarkers in active SLE

**Figure 5:**
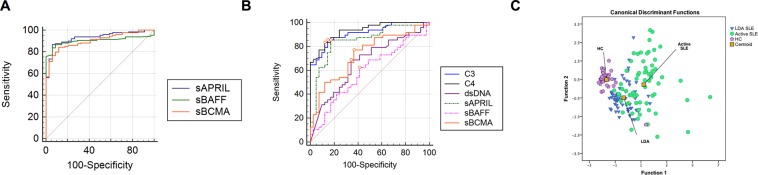
Evaluation of serum B cell proliferation factor levels as potential biomarkers of active SLE. Areas under the receiver operating characteristic (ROC) curve (AUCs) for prediction models discriminating (**A**) SLE patients and HCs or (**B**) clinically active disease and LDA. ROC curves are shown for sBAFF (dotted pink line), sAPRIL (dotted green line), sBCMA (orange line), C3 (blue line), C4 (black line) and anti-dsDNA antibodies (purple line). Youden’s J index (open orange circles) (**C**) Discriminant analysis using cytokine levels to classify active SLE patients (green circles), LDA SLE patients (blue triangles), and HCs with no personal or family history of autoimmunity (purple pentagons). Two canonical discriminant functions, function 1 and function 2, were generated based on their individual standardized coefficients. There is clear discrimination among the 3 groups, and the model predicts group membership with 80.3% accuracy. Full squares represent the group centroid. BCMA: B-cell maturation antigen; BAFF: B-cell activating factor; APRIL: a proliferation-inducing ligand.

To assess the sensitivity and specificity of sBCMA, sBAFF, and sAPRIL in the serum as biomarkers for SLE, we performed receiver operating characteristic (ROC) curve analysis (Fig. 5A). The areas under the curve (AUCs) for sAPRIL, sBAFF and sBCMA were high (0.971, 0.905 and 0.904, respectively). At a cutoff value of 22.40 ng/mL (likelihood ratio (LR): 16.34), sAPRIL showed a high sensitivity (96.12%) and specificity (94.12%) for discrimination between HCs and SLE patients, while sBAFF showed lower sensitivity (82.2%) but higher specificity (94.1%) at a cutoff of 1.195 ng/mL (LR: 13.97). The cutoff value of 30.88 ng/mL (LR: 7.18) showed moderate sensitivity (84.5%) and sensibility (88.24%) for sBCMA.

Next, we compared the ability to differentiate patients with LDA vs AD using ROC curve analysis of soluble B cell factors (Fig. 5B) and conventional biomarkers used to monitor SLE disease activity including anti-dsDNA antibodies, C3 and C4. We found that the AUC for sBAFF levels was low (0.590), and ROC curve analysis showed that sAPRIL and sBCMA produced higher AUCs than anti-dsDNA antibodies (Table 2). On the other hand, the AUC of sAPRIL levels was 0.847, while the sBCMA AUC was 0.730. We calculated the sensitivity and specificity of the markers at different cutoff levels and found the sensitivity and specificity to be highest at cutoffs of 2.31 ng/ml for sBAFF (sensitivity: 62.5%; specificity: 60.5%), 51.17 ng/ml for sAPRIL (sensitivity: 83.3%; specificity: 81.4%) and 28.01 ng/ml for sBCMA (sensitivity: 77.1%; specificity: 62.8%). To determine whether a combination of either cytokine with the soluble receptor BCMA would improve diagnostic specificity, the combined specificity was calculated. To achieve this, we calculated the combined sensitivity as follows [(sCytokine)sens * (sBCMA)sens]. The combined sensitivity was 48.18% for sBAFF and sBCMA and 64.22% for sAPRIL and sBCMA.

**Table 2:** Performance of biomarkers to predict active SLE.

Biomarker	AUC	95% C.I.	Cut-off point	Youden’s J index	Sensitivity	Specificity	LR +
Anti-dsDNA antibodies	0.675	0.569 to 0.770	>23.36 IU/mL	0.3619	68.7	67.4	2.11
C3	0.911	0.832 to 0.961	≤120.2 mg/dL	0.7078	85.4	85.4	5.84
C4	0.932	0.859 to 0.975	≤24.2 mg/dL	0.7114	83.3	87.8	6.83
sBCMA	0.730	0.627 to 0.818	>28.01 ng/mL	0.3987	77.1	62.8	2.07
sBAFF	0.590	0.482 to 0.692	>2.31 ng/mL	0.2297	62.5	60.5	1.58
sAPRIL	0.847	0.757 to 0.914	>51.17 ng/mL	0.6473	83.3	81.4	4.48

AUC: area under a receiver operating characteristic (ROC) curve, C.I.: confidence interval, LR: likelihood ratio.

The data were also subjected to multivariate statistical analyses to establish whether a set of these variables (soluble B cell factors) can be used to distinguish active disease and LDA groups. Discriminant function analysis was limited to the variables sAPRIL and sBCMA and included 63 LDA SLE patients, 66 AD SLE patients, and 34 HCs. This analysis revealed three clusters in cytokine and soluble receptor distribution that were separated and distinct only for the active SLE patients and healthy controls (Fig. 5C), while the separation of the cluster containing the LDA SLE patients was less reliable. The model predicted group membership based on these discriminant functions with an overall accuracy of 80.3%. The validity of the variables in differentiating the groups was supported by Wilks’s lambda coefficient (0.385), and the F-test of Wilks’s lambda was significant (*p* < 0.05).

## Discussion

The BAFF/APRIL system has emerged as a critical player in pathologies associated with impaired B cell function and autoimmune diseases such as SLE, which is characterized by the presence of autoantibodies highly specific for target tissues.

The participation of BCMA in SLE has remained elusive, and some authors have reported increased expression of mBCMA on CD19+ B cells from SLE patients compared with those from HCs^25,31,32^. Contrary to those results, in a previous study by our group and the present study, we found diminished expression rates for the mBCMA receptor on CD19+ B cells from SLE patients, as this expression was almost null in patients with severe disease. Moreover, the receptor expression rate inversely correlated with disease activity^33^. The results were in concordance with those reported by Zhao *et al*., who found that the percentage of CD19+ mBCMA+ cells negatively correlated with the titres of anti-dsDNA antibodies and the SLEDAI, even though they reported increased BCMA expression in SLE patients^32^. On the other hand, we observed that antigen-experienced B cells, such as SLE plasma cells, expressed mBCMA at a relatively high rate^32,34^. This could be explained mainly because these cells are relatively dependent on BCMA at this stage^24^, since the main functions of the receptor are related to immunoglobulin class switching and plasma cell maintenance^26^. Recently, a group of researchers found that BCMA deficiency accelerated the development and exacerbation of disease when developing a BCMA knockout model in a strain susceptible to lupus (*Nba2*)^35^. These findings were unanticipated given that BCMA^-/-^ mice have normal B cell development and immunoglobulin serum concentrations^36^. The same research group later reported that the deficiency in BCMA expression in T cells promoted the expansion of LT_FH_ cells in the spleen, accompanied by increased production of IFN-γ and antibodies, apparently through BAFF-R^37^. In this study, the percentage of BCMA +  CD3+ cells was diminished, and the serum levels of IFN-γ were elevated in SLE patients and correlated with sBCMA expression. The results reported by Coquery *et al*. suggest that the balance between BAFF-R and BCMA in T cells works to regulate immune tolerance.

Based on these observations, we aimed to investigate whether soluble BCMA levels were related to clinical features in SLE patients. The first report on sBCMA was in MM patients^28^, and those with progressive disease maintained higher serum sBCMA levels than those with stable disease. Therefore, sBCMA was proposed as a biomarker for monitoring disease status in MM. The mechanism that allows the release of BCMA remained unknown until recently, when Laurent *et al*. reported that the protease γ-secretase is responsible for BCMA shedding from the plasma membrane. The study revealed a novel mechanism for γ-secretase cleavage activity, as the naturally short extracellular domain of BCMA, comprising only one CRD, allowed the release of the soluble form without truncation^27^. As cellular immune and inflammatory events regulated by signalling cascades are tightly regulated, the presence of soluble forms of receptors (termed decoy receptors) constitutes a parallel regulatory axis for immunomodulatory pathways^38^.

We found increased sBCMA levels in SLE patients that correlated with disease activity. These results are in concordance with those of Laurent *et al*., who also quantified serum sBCMA levels in a small sample of patients with autoimmunity and found that SLE patients showed sBCMA serum levels correlated with SLEDAI^27^. Additionally, we observed that serum sBCMA levels were associated with anti-dsDNA antibody positivity, as had been reported by Vincent *et al*.^29^.

It is important to note that even though BCMA expression on the cell surface is low; within autoimmune hosts, BCMA is expressed in the Golgi apparatus of plasma cells^39^. Additionally, the display of BCMA on the surface of human pDCs after TLR7/TLR9 engagement leads to the release of sBCMA. Because the expression of BCMA is not restricted to B cells, it is important to consider the release of sBCMA by cellular sources such as pDCs in response to TLR stimulation in blocking therapies targeting the BAFF-APRIL system in autoimmune diseases such as SLE^40^. These mechanisms contribute to the increased dependency on BAFF/APRIL-mediated survival mechanisms to promote apoptosis in autoreactive B cells in SLE.

On the other hand, sBAFF and sAPRIL levels were increased in SLE patients in concordance with previous studies^6–11^, and both correlated with disease activity evaluated by the SLEDAI-2K. It is important to note that sAPRIL displayed a stronger correlation than did sBAFF. Previous reports have shown the associations of sBAFF and sAPRIL levels with disease activity and musculoskeletal, haematological and renal manifestations in SLE^6,8–10,33^. Nevertheless, the controversy regarding the utility of both cytokines as disease activity biomarkers remains, since other authors have reported a lack of associations^11,41–44^.

In addition, we observed decreases in sBCMA and sBAFF expression in SLE patients who achieved LDA after 6 months, and we hypothesized a possible regulatory role for sBCMA as a decoy receptor for BAFF. In the measurement of B cell factor levels in cross-sectional studies, mixed results have been reported. A longitudinal analysis of 87 SLE patients in an Australian cohort revealed no significant association between the serum sBCMA level at baseline and clinical parameters over time^29^. A study found a reduction in the serum BAFF levels of SLE patients with no change in the SLEDAI-2K between visits; however, baseline serum BAFF and APRIL concentrations did not associate with subsequent changes in disease activity^11^. Another study found no association between serum BAFF level changes and RA patient relapse^45^, which could indicate that the decoy capacity of sBCMA can be overridden if BAFF is produced at abnormally high levels^37^.

It is relevant to mention that SLE patients who did not achieve remission had increased sBAFF levels after six months, despite pharmacological treatment. This finding is similar to the results of Vincent *et al*., who found that SLEDAI-2K > 3 SLE patients exhibited increased serum BAFF expression^11^. These findings suggest that the behaviour of serum BAFF when disease activity increases differs from that when disease activity remains stable. Furthermore, as active SLE patients show sBAFF levels above 2 ng/mL, we agree with the findings of Petri *et al*., who established 2 ng/mL sBAFF as a cutoff to predict peaks of reactivation of moderate to severe disease^8^.

Whether endogenous BAFF can signal through BCMA *in vivo* remains inconclusive, but it will undoubtedly depend on avidity effects. An *in vitro* analysis of the BAFF-BCMA interaction suggests that multimerized forms of soluble BAFF (60-mer) as well as clustering of membrane-bound BCMA or BAFF have relatively high avidity effects^16^. Soluble BCMA-Ig is capable of effectively neutralizing BAFF activity *in vivo* and *in vitro* and decreasing B cell numbers^7,46^.

However, as BCMA binds APRIL with high affinity, the APRIL–BCMA axis is considered to be the responsible factor for B cell differentiation at later stages or at least partially reduces BAFF dependence^16^. sBCMA and sAPRIL could be valuable biomarkers for disease activity, as both showed higher sensitivity and specificity than sBAFF in discriminating active SLE patients.

It has been found that *in vitro*, APRIL induces the upregulation of the expression of numerous costimulatory molecules in B cells, such as CD40^47^, which significantly increases the presentation of antigens. This effect is managed by BCMA, not by TACI or BAFF-R, due to the ability of BCMA to activate both the NF-κB and JNK pathways, which are necessary pathways for the increase in antigen presentation^19^. TNF receptor-associated factor (TRAF) 2, TRAF5 and TRAF6 interact with the cytoplasmic region (amino acids at position 119–143) of BCMA, and these associations are required for NF-κB activation^23^. The association of BCMA and TRAF2 also activates MAPK pathways, principally the ERK pathway, through the downstream transcription factor Elk-1, leading to the activation of target genes that promote cell survival and proliferation^48^. In general, BCMA promotes the survival of plasmablasts and plasma cells and therefore has a predominant role in humoural immunity^37^. *In vitro*, sBCMA acts as a decoy receptor to restrict the APRIL-mediated survival of activated primary B cells^27^.

Altogether, these study results show that the participation of BCMA in SLE pathogenesis is more critical than previously thought, and we consider BCMA particularly relevant for current clinical trials targeting the cytokines BAFF/APRIL. However, our study has some limitations to consider, such as the reduced size of the prospective sample cohort and the fact that we evaluated only sBCMA. The simultaneous evaluation of the soluble receptors sBAFF-R^49^ and sTACI^50^ could provide new insight into the biological mechanisms of the BAFF/APRIL system. Additionally, we consider it essential to evaluate the activity of γ-secretase and possibly the cellular sources of both the enzyme and soluble decoy receptors in SLE patients.

Although the function of sBCMA in autoimmune diseases has been poorly studied, we show its possible role in the regulation of SLE. sBCMA probably acts as a natural decoy receptor to neutralize the functions driven through its ligands, particularly sAPRIL. In summary, a more comprehensive study is needed to elucidate the roles of BAFF/APRIL soluble decoy receptors, not only sBCMA, in the immune tolerance regulation that occurs in a complex disease such as SLE.

## Methods

### Patients and healthy controls

The study included one hundred and twenty-nine patients with SLE fulfilling the 1997 revised American College of Rheumatology criteria who were recruited from the Department of Rheumatology and Immunology at West Medical Hospital, Mexico. Additionally, we included 34 unrelated subjects from the general population; these subjects were blood donors with no history of autoimmune or chronic inflammatory disease and were used as sex- and age-matched healthy controls (HCs). At the time of sampling in all SLE patients, the rheumatologist determined scores for the Mexican version of the Systemic Lupus Erythematosus Disease Activity Index (Mex-SLEDAI)^51^ and Systemic Lupus International Collaborating Clinics index (SLICC)^52^. A Mex-SLEDAI score >2 was considered a marker of active disease^53^. Patients who showed only mild manifestations, such as leukopenia (1 pt), lymphopenia (1 pt), or fever and fatigue (1 pt), and did not require adjusted treatment were classified as having LDA. We considered all patients with other manifestations, including serositis (2 pts), mucocutaneous (2 pts), arthritis (2 pts), myositis (3 pts), haemolysis/thrombocytopenia (3 pts), vasculitis (4 pts), renal manifestations (6 pts) and neurological manifestations (8 pts), as having active SLE. SLE clinical disease activity was measured by the Systemic Lupus Erythematosus Disease Activity Index 2000 (SLEDAI-2K)^54^. The low disease activity (LDA) group was defined by a SLEDAI-2K index ≤4 and allowed treatment with HCQ and prednisolone (≤7.5 mg/day)^55,56^. Seventeen of the SLE patients were included in a prospective analysis (intervals of ~six months), and the change in disease activity was evaluated by the Mex-SLEDAI, the SLEDAI-2K and the British Isles Lupus Assessment Group (BILAG) index^57^. The SLE patients included in the study received standard-of-care pharmacological treatment that did not include biological agents.

### Ethics statement

This study was approved by the Ethics and Research Committee of West Medical Hospital (No. 561/18). Written informed consent was obtained from the participants before inclusion in the study. All clinical investigations in this study were conducted according to the principles expressed in the Declaration of Helsinki.

### Laboratory assessments

A complete blood panel (CELL-DYN 3500 R; Abbott Diagnostics, Lake Forest, IL, USA) and the erythrocyte sedimentation rate (ESR) determined using Wintrobe’s method were analysed in both groups. Results for anti-nuclear antibodies (ANAs), anti-Ro, anti-La, and anti-RNP antibodies were taken from patient medical records. The serum of SLE patients was collected at the time of enrollment, aliquoted and stored at −20 °C until use to determine anti-dsDNA antibodies, C3 and C4 concentrations. Repeated freeze/thaw cycles were avoided^58,59^. The levels of anti-dsDNA antibodies (Anti-dsDNA IgG, Cat. ORG604, ORGENTEC Diagnostika; Mainz, DE), complement C3 (Human C3, Cat. ab108823, Abcam plc; Cambridge, UK) and complement C4 (Human C4, Cat. ab108824, Abcam plc) were determined by ELISA.

### Flow cytometry

PBMCs were separated from the buffy coats of 20 SLE patients and 20 HCs by Histopaque-1077 (Merck KGaA; Darmstadt, Germany) density gradient centrifugation. Membrane BCMA expression was determined with a BD FACSAria I cytometer (BD Biosciences, San Jose CA, USA) using the appropriate combination of allophycocyanin (APC)-conjugated anti-human BCMA (Cat. FAB193A; R&D Systems, Minneapolis, MN, USA), APC/Cy7-conjugated anti-CD3 (Cat. 344818; BioLegend, San Diego, CA, USA), PerCP-conjugated anti-CD19 (Cat. 302228; BioLegend, San Diego, CA, USA), FITC-conjugated anti-CD27 (Cat. 302806; BioLegend, San Diego, CA, USA), and PE/Cy7-conjugated anti-CD38 (Cat. 303516; BioLegend, San Diego, CA, USA) antibodies. The appropriate isotype and fluorescence minus one (FMO) controls were used to adjust for background fluorescence and perform gating, and the results are reported as the percentage (%) of expression and the median fluorescence intensity (MFI) for CD3+, CD19^+^, and CD19^+^CD27^+^CD38^+^ (plasma cells) populations. Data were analysed using FlowJo v.9 (BD, Franklin Lakes, NJ, USA). The gating strategy is shown in Fig. 1A.

### Soluble receptor and cytokine serum level quantification

The serum of SLE patients was collected at the time of enrolment, aliquoted and stored at −20 °C until use to determine cytokine concentrations. Repeated freeze/thaw cycles were avoided^58,59^. Levels of sBCMA (Human TNFRSF17, Cat. ab213840, Abcam plc; Cambridge, UK) and the cytokines sBAFF (Human TNFSF13B, Cat. DBLYS0B) and sAPRIL (Human TNFSF13 DuoSet, Cat. DY884B) in the serum were measured by ELISA (R&D Biosystems; Minneapolis, MN, USA) following the manufacturer’s protocols. The absorbance at 450 nm and 540 nm was determined with the plate reader Multiskan GO spectrophotometer (Thermo Fisher Scientific Inc; Vantaa, Finland). The detection limits were 10 pg/mL for sBCMA, 6.44 pg/mL for BAFF and 31.3 pg/mL for APRIL. The serum levels of IFNγ, IL6, IL10, IL4 and TNFα were determined with a Bio-Plex Pro Human cytokine panel kit (Cat. 171AA001M; Bio-Rad Laboratories, Hercules, CA, USA) following the recommendations of the manufacturer. The beads were read on a Bio-RAD MAGPIX® instrument.

### Data analysis

The Shapiro-Wilk test was used to assess the normality of data distributions. Categorical variables are presented as absolute values and percentages, and continuous variables are expressed as medians and 25th-75th percentiles. Differences among groups were compared by the Kruskal-Wallis test, followed by Dunn’s multiple comparisons test if more than two subgroups were compared. For comparisons of two groups, the Mann-Whitney test was applied. To determine correlations between parameters, Spearman’s rank correlation coefficient was performed, followed by the Bonferroni correction to control for multiple comparisons. Changes in parameters following treatment were assessed using Wilcoxon’s matched‐pairs signed‐rank test. Receiver operator characteristic (ROC) curve analysis was performed to assess the value of serum B cell proliferation markers in differentiating between LDA and AD SLE patients with MedCalc 19.1.6 software (MedCalc Software Ltd, Ostend, Belgium). Statistical analyses were performed using SPSS v.25 (IBM Corporation; Armonk, NY, USA) or GraphPad Prism 8.0 (GraphPad Software Incorporation; La Jolla, CA, USA) software packages. A *p*‐value ≤ 0.05 was considered significant.

## Supplementary information

Supplementary Table S1.

## Data Availability

The datasets generated and/or analysed during the current study are available from the corresponding author upon reasonable request.
