# Supplementary material for: Analysis of the receptor BCMA as a biomarker in systemic lupus erythematosus patients
Source: Sci Rep. 2020 Apr 10;10:6236. doi: 10.1038/s41598-020-63390-0 (PMC7148319; doi:10.1038/s41598-020-63390-0)
Supplement: Supplementary file 1 — Supplementary Table S1. [file 41598_2020_63390_MOESM1_ESM.pdf]

Analysis of the receptor BCMA as a biomarker in systemic lupus erythematosus patients

Diana Celeste Salazar-Camarena[1], Claudia Azucena Palafox-Sánchez*[1], Alvaro Cruz[1], Miguel Marín-Rosales[2], José Francisco Muñoz-Valle[1].

[1]Research Institute of Biomedical Sciences, University Center for Health Sciences, 44340, University of Guadalajara, Mexico.

[2]Department of Rheumatology, West Medical Hospital, Ministry of Health, Zapopan, 45170, Mexico.

No.	Age	Disease duration, years	BILAG-2004 Baseline		BILAG-2004 Follow up		Mex-SLEDAI		SLEDAI-2K		SLICC		Baseline		Follow up	
			Domains (Category)	TOTAL	Domains (category)	TOTAL	Baseline	Follow up	Baseline	Follow up	Baseline	Follow up	Treatment	Steroid dosage	Treatment	Steroid dosage
1*	49	26	HM(C), RN(C)	2	HM(C), RN(C)	2	2	2	2	0	6	6	AZ	_	AZ	_
2*	32	1	RN(B), HM(C)	9	RN(B), HM(D)	8	7	2	12	4	0	2	PD, AM,AZ, CP	10	PD, AM,AZ, CP	10
3*	20	2	MC(D), MS(D), GI(D) RN(C), HM(C)	2	MC(D), MS(D), GI(D) RN(D), HM(C)	1	7	2	12	3	0	0	PD, AM, AZ	10	PD, AM, AZ	7.5
4*	17	3	MC(D), MS(D),CR(D), RN(D), HM(D)	0	MC(D), MS(D),CR(D), RN(D), HM(D)	0	1	1	2	2	1	1	AM, AZ	_	AM, AZ	_
5*	52	10	CN(D), MS(D),GI(D), RN(B), HM(D)	8	CN(D), MS(D),GI(D), RN(B), HM(D)	8	0	0	3	2	2	2	PD, AM, AZ	5	PD, AM, AZ	5
6*	28	9	MC(C), NE(D),	1	MC(B), NE(D), MS(C)	9	0	3	0	2	1	1	AM	_	AM	_
7*	17	1	MC(D), NE(D), MS(D), RN(D), HM(D)	0	MC(B), NE(D), MS(D), RN(D), HM(C)	9	0	3	2	2	0	0	PDN, AM, AZ, CP	25	PDN, AM, AZ	5
8*	30	2	HM(D)	0	HM(D), MS(C)	1	0	2	0	4	0	0	PDN, AM, AZ	15	PDN, AZ	7.5
9	21	1	CN(D), MS(D) CR(D) HM(D)	0	CN(D), MS(B) CR(D) HM(D)	8	0	2	2	6	0	0	PD, AM,MT	5	PD, AM,MT	5
10	33	0.5	MC(C), CR(B), RN(C),HM(B)	18	MC(C), CR(D), RN (B), HM(C)	10	10	2	8	6	0	1	PD	75	PD. MT	5
11	29	10	MC(B), MS(D), RN(A), HM(C)	21	MC(C), MS(D), RN(A), HM(B)	14	4	8	12	12	1	2	CP, PD, AM	2.5	PD, MF, AZ	25
12	29	1	MC(D), MS(D), RN(A), HM(C)	13	MC(D), MS(D), RN(A), HM(C)	13	6	7	8	8	1	2	PD, AZ, CP	15	PD, AM, AZ, CP	15
13	25	1.6	MC(B), MS(B),RN(A), HM(C)	29	MC(B), MS(D),RN(B), HM(C)	17	11	9	24	16	0	0	PD, AM, AZ, CP	2.5	PD, AM, AZ, CP	5
14	28	2	MC(B), MS(B), RN(A), HM(C)	29	MC(D), MS(D), RN(A), HM(C)	13	9	8	12	14	0	0	PD, AM, AZ	50	PD, AM, CP, MF	50
15	34	2	CN(D), MC(B), MS(C), RN(C)	10	CN(D), MC(D), MS(D), RN(B), HM(C)	9	2	7	6	8	0	1	PDN, AM, AZ, CP	10	PDN, AM, AZ, CP	5
16	41	12	MC(C), MS(D), RN(B), HM(D)	9	MC(C), MS(D), RN(B), HM(D)	9	6	3	6	4	2	2	PDN, AZ, CP	10	PDN, AZ	10
17	23	7	RN(A), HM(C)	13	RN(A), HM(C)	9	9	7	8	6	1	1	PD, AZ, CP	20	PD, AZ, CP	15

Supplementary Table S1. Clinical features in the SLE cohort. *SLE patients that maintain or reached LDA at follow-up. CN: constitutional, CR: cardiorespiratory, GI: gastrointestinal, HM: hematological, MC: mucocutaneous, MS: musculoskeletal, NE: neurological, RN: renal, AZ: azathioprine, PD: prednisone, CQ: chloroquine, CP: cyclophosphamide, MP: mycophenolate, MT: methotrexate.

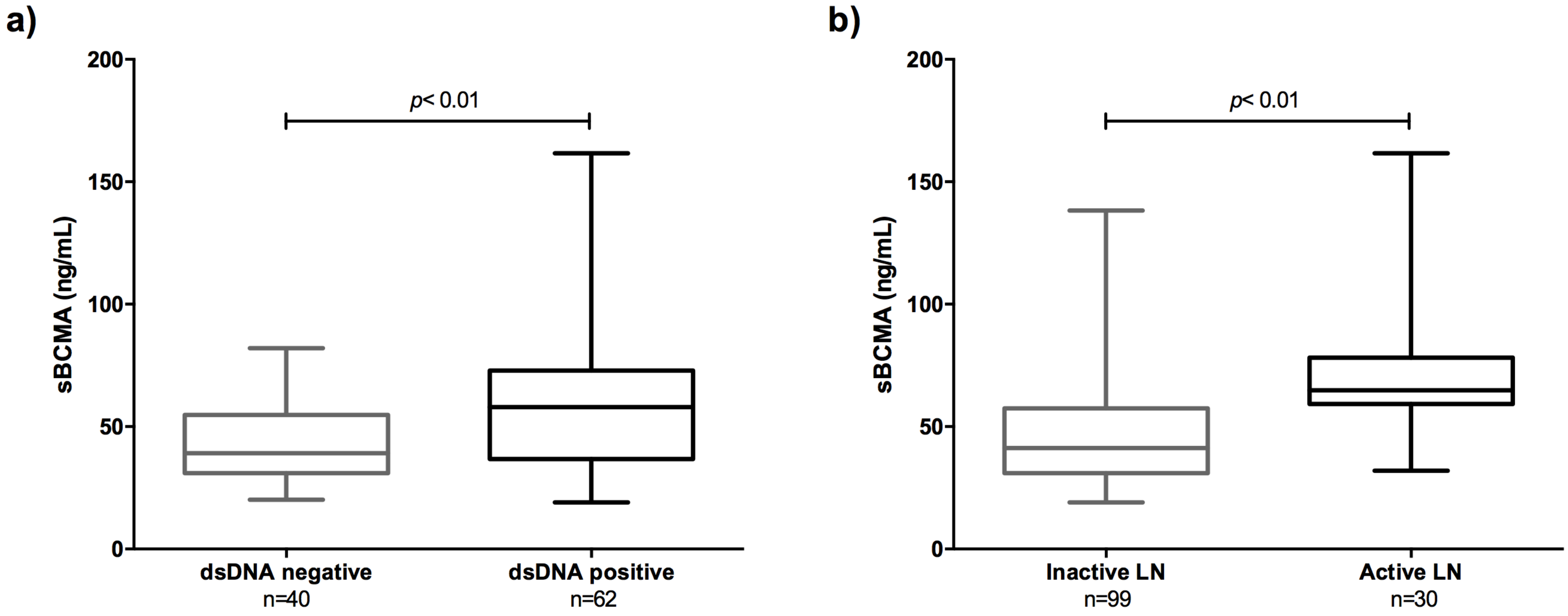

Supplementary Fig. S1
Serum sBCMA levels according clinical features in SLE patients. (a) Anti-dsDNA antibody positivity. (b) sBCMA levels in inactive lupus nephritis vs active lupus nephritis. NL: lupus nephritis.
